# Plasma metabolomic and lipidomic signatures characteristic of treatment non-response in rheumatoid arthritis

**DOI:** 10.3389/fimmu.2026.1787287

**Published:** 2026-06-04

**Authors:** Jiani Chen, Tong Sun, Weili Luo, Chengyang Cao, Jiaqin Xu, Jiaxi Chen

**Affiliations:** 1 Taizhou Hospital of Zhejiang Province Affiliated to Wenzhou Medical University, Linhai, China; 2 Hangzhou Medical College, Hangzhou, China

**Keywords:** biomarkers, lipidomics, metabolomics, rheumatoid arthritis, treatment response

## Abstract

**Objectives:**

This study aimed to investigate plasma metabolomic and lipidomic profiles in rheumatoid arthritis (RA) to identify potential biomarkers for distinguishing treatment responses.

**Methods:**

Plasma samples were collected from 106 RA patients and 10 healthy controls, with 30 RA samples selected based on predefined inclusion criteria. Using liquid chromatography–mass spectrometry (LC-MS)-based untargeted metabolomics and lipidomics analysis, a total of 2,279 metabolites and 2,987 lipids were tentatively annotated.

**Results:**

Through rigorous statistical evaluation (variable importance in the projection (VIP) > 1 and false discovery rate (FDR) < 0.05), 22 metabolites and lipids were found to be positively associated with RA risk based on logistic regression analysis. These were further refined into 12 core features using least absolute shrinkage and selection operator (LASSO) regression. The 12 core features identified in the treatment non-response group included lipids Cer(d18:1/16:0), PS(16:0/20:0), and Palmitic acid; metabolites Mimosine and D-Xylose; drug-related metabolites Dihydralazine, Tridihexethyl, Acyclovir monophosphate, Indoline, and Melleolide; and pollution-related metabolites Norcotinine and 2-Chloro-1-(chloromethyl)ethyl carbamate. Machine learning models utilizing these features showed promising preliminary discriminatory performance in internal cross-validation, achieving area under the curve (AUC) values exceeding 0.90 in this cohort, suggesting the potential utility of these candidate biomarkers in differentiating treatment-responsive from non-responsive RA patients. Additionally, the identified features showed significant correlations with high disease activity, with preliminary evidence of potential associations across different stages of disease progression.

**Conclusion:**

The findings suggest significant metabolic and lipidomic alterations in RA, and indicate that these candidate biomarkers may serve as preliminary indicators of treatment response.

## Introduction

Rheumatoid Arthritis (RA) is a common immune-mediated inflammatory disease, whose clinical manifestations are mainly characterized by persistent pain, swelling, and stiffness of synovial joints. Early symptoms mostly involve the joints of the hands and feet, especially the metacarpophalangeal joints and metatarsophalangeal joints (1). With disease progression, persistent inflammation invades the bone tissue around the joints, leading to irreversible damage to articular cartilage, bones, and joint capsules, which can eventually cause joint deformity and dysfunction (2). Globally, the prevalence of RA in adults is approximately 0.5% to 1.0% (3). Similar to many other autoimmune diseases, the treatment of RA still faces considerable challenges, and the existing clinical intervention methods have limitations. Clinical treatment adopts a target-oriented strategy, whose core is to select an appropriate treatment plan to achieve disease remission or reduce disease activity. Symptomatic treatment mainly includes nonsteroidal anti-inflammatory drugs (NSAIDs) and glucocorticoids; disease-modifying treatment is mainly based on disease-modifying antirheumatic drugs (DMARDs), which are mainly divided into three categories: conventional synthetic DMARDs (csDMARDs), biological DMARDs (bDMARDs), and targeted synthetic DMARDs (tsDMARDs) (4, 5).

In clinical practice, csDMARDs serve as the standard first-line therapeutic regimen for patients with RA. For individuals who are intolerant to first-line csDMARDs therapy or fail to achieve adequate clinical response, bDMARDs or tsDMARDs will be considered as subsequent treatment options (6). The emergence of these biological agents and targeted therapeutics has profoundly altered the disease course of a subset of RA patients (2). Among these biological agents, TNF-α inhibitors represent the most widely applied first-line biological therapy in the current management of RA. In the clinical management of rheumatoid arthritis, TNF-α inhibitors are commonly classified into two types: monoclonal antibodies and receptor fusion proteins. Monoclonal antibodies include golimumab, adalimumab, and infliximab, whereas receptor fusion proteins primarily consist of etanercept and its biosimilars. These inhibitors effectively block the binding of TNF-α to its receptor and are widely utilized in patients who do not achieve adequate responses to traditional DMARDs (7, 8). While some patients may attain remission, a substantial number remain unable to achieve immune homeostasis or clinical improvement due to the heterogeneity of the disease (9, 10). Therefore, there is an urgent need for non-invasive diagnostic methods that offer high sensitivity and specificity to facilitate timely prognostic assessments and disease management, potentially leading to improved clinical outcomes.

The extent of metabolic changes and the types of metabolites observed may serve as valuable markers for cytokine-mediated inflammatory processes in RA (11, 12). Altered metabolic profiles could be instrumental in predicting disease development in patients with early arthritis and evaluating treatment responses (13). The metabolomic signature of RA is dynamically altered in response to therapeutic intervention, and such metabolic changes are highly correlated with clinical improvements in patients (14). Similarly, lipids are critical metabolites that function as energy sources, membrane constituents, and signaling molecules. Lipids are significant in the inflammatory response as signaling molecules (15, 16). Therefore, liquid chromatography–mass spectrometry (LC-MS)-based metabolomics and lipidomics analyses are recognized as powerful tools for discovering potential prognostic biomarkers during RA treatment (17). These advanced techniques facilitate comprehensive profiling of metabolites and lipids, providing insights into the underlying biochemical processes and inflammatory pathways that could ultimately enhance RA patients’ management and therapeutic strategies.

Current research in the field of RA omics has seldom focused on patients with poor treatment outcomes, particularly those receiving TNF-alpha therapy. Therefore, this study was conducted to perform metabolomics and lipidomics analyses using plasma samples from 30 RA patients and 10 healthy controls (HC), based on LC-MS techniques. By stratifying the enrolled patients, particular attention was given to those with inadequate treatment responses. Additionally, a prognostic diagnostic model was developed through machine learning, which was validated using four internal validation methods (e.g., Support Vector Machine (SVM, Linear), Random Forest, Partial Least Squares, and Logistic Regression). This study aims to enhance the understanding of biochemical alterations associated with treatment non-response and to identify potential biomarkers that may facilitate the management of RA patients who do not respond adequately to conventional therapies.

## Materials and methods

### Study cohort

Peripheral blood samples were initially collected from 106 patients with RA and 10 healthy controls (HC). All RA patients initially included fulfilled the diagnostic criteria established by the American College of Rheumatology (ACR) or the European League Against Rheumatism (EULAR) guidelines (18, 19). After rigorous screening according to the unified grouping stratification criteria, only 30 RA patients were finally enrolled in the subsequent omics analysis. The screening and exclusion workflow was strictly implemented based on standardized inclusion and exclusion criteria for RA patients. Eligible patients were required to be aged ≥18 years with a disease duration of at least 6 weeks, and to have received treatment with at least one DMARD. In addition to the general exclusion conditions (diagnosis of any autoimmune disease other than RA, severe chronic or current infections, cancer, pregnancy or lactation), individuals with incomplete clinical data, missing DAS28 scores or treatment follow-up information, as well as those with irregular and non-standard antirheumatic medication regimens were further excluded to ensure the clinical homogeneity and reliability of the enrolled cohort. The detailed patient screening workflow is presented in **Supplementary Figure 1**. HC were required to meet the inclusion criteria of being ≥18 years old with normal results from recent liver and kidney function tests. The exclusion criteria for healthy controls were (1): a diagnosis of any autoimmune disease (2), a history of severe chronic infections or the presence of any current infection (3), diagnosis of cancer, and (4) pregnancy or lactation. Venous blood samples were obtained from all participants in the morning prior to breakfast. Following collection, blood samples were centrifuged to isolate plasma, which was stored at −80 °C until further analysis.

This study was conducted in accordance with the principles outlined in the Declaration of Helsinki and was approved by the Medical Ethics Committee of Enze Hospital, Taizhou Enze Medical Center (Group) (approval number: K20240709). This study is a retrospective study and all research procedures were conducted without direct contact with patients. The plasma samples and corresponding clinical data of all participants (RA patients and healthy controls) were obtained from the residual biological specimen repository and existing clinical records of our hospital, and no new sample collection or related experimental operations were performed by the research team for this study.

For the healthy controls, all specimens were derived from the residual samples of the physical examination center of our hospital, rather than subjects specifically recruited for this study. The healthy status of the control group was exclusively determined based on the results of routine clinical examinations in the physical examination report (including complete blood count, liver and kidney function, and rheumatism-related serological indicators), with no additional special screening or enrollment procedures implemented for healthy control recruitment.

### Liquid chromatography–tandem mass spectrometry analysis

Sample preparation for both metabolomics and lipidomics involved thawing samples at 4 °C and subjecting them to specific extraction protocols: metabolomics samples were treated with methanol/acetonitrile (1:1), while lipidomics samples were mixed with methanol/water (2:1) and methyl tert-butyl ether. Both were vortexed, centrifuged, and vacuum dried before storage at −80 °C (20). Chromatographic separation was performed using a Nexera UHPLC LC-30A system with distinct mobile phases for positive and negative ion modes and tailored elution gradients for each analysis. Following separation, a Q Exactive HF-X mass spectrometer was used for mass spectrometric analysis, optimizing parameters for parent and fragment ion scanning in both mode (21). Specific parameters and methods are detailed in the supplementary materials.

Raw metabolomic and lipidomic features acquired under positive (POS) and negative (NEG) ionization modes were subjected to systematic data preprocessing. Firstly, features with a missing value ratio greater than 50.00% across all experimental samples were removed. Secondly, background noise was eliminated by discarding features with relative standard deviation (RSD) > 30.00% in quality control (QC) samples. Remaining missing values were imputed with one-tenth of the minimum intensity value of the corresponding feature.

For compound identification and functional annotation, candidate compounds were matched according to accurate molecular weight, MS/MS fragmentation patterns, mass error (ppm), and isotope similarity. All annotated molecules were further assigned with multiple chemical and database information, including formula, adduct forms, InChIKey, HMDB ID, KEGG ID, ChEBI ID, PubChem compound ID, CAS registry number, SMILES structure, as well as hierarchical classification information (kingdom, super_class, class, sub_class).

Notably, all metabolites and lipid species reported in this study are tentatively identified based on database spectral matching and computational annotation. No commercial chemical standards were applied for individual molecular validation. Therefore, structural isomers and stereoisomers with identical m/z and similar MS/MS profiles could not be further distinguished in the current workflow.

### Data processing and statistical analysis

Quantitative data for metabolites and lipid compounds were normalized using z-score transformation. Partial least squares discriminant analysis (PLS-DA) and variable importance in projection (VIP) were conducted using the R package “ropls.” Differences were assessed through analysis of variance (ANOVA), with results adjusted for multiple testing using false discovery rate (FDR) correction. Trend clustering analysis was performed utilizing the Mfuzz R package (22). Correlation coefficients between metabolites, lipids, and clinical data were analyzed using Spearman’s rank correlation test. Kyoto Encyclopedia of Genes and Genomes (KEGG) pathway analysis was conducted using MetaboAnalyst 6.0 (www.metaboanalyst.ca) (23). Univariate analysis was carried out using standard logistic regression as the primary method. To verify the stability of the odds ratio (OR), sensitivity analysis was performed using Firth penalized regression. Stability selection was executed using least absolute shrinkage and selection operator (LASSO) regression with 10,000 cycles of cross-validation (24). A classification model was constructed using Random Forest optimization, incorporating parameter grid search combined with 10×5 cross-validation, and the performance of models, including SVM (Linear) (25), PLS (26), and logistic regression, was compared. The discriminative ability was assessed through receiver operating characteristic (ROC) curve analysis. All statistical analyses were performed using R software (version 4.3.2).

## Results

### Study design and analytical approach for plasma metabolomics and lipidomics in rheumatoid arthritis

**Figure 1:**
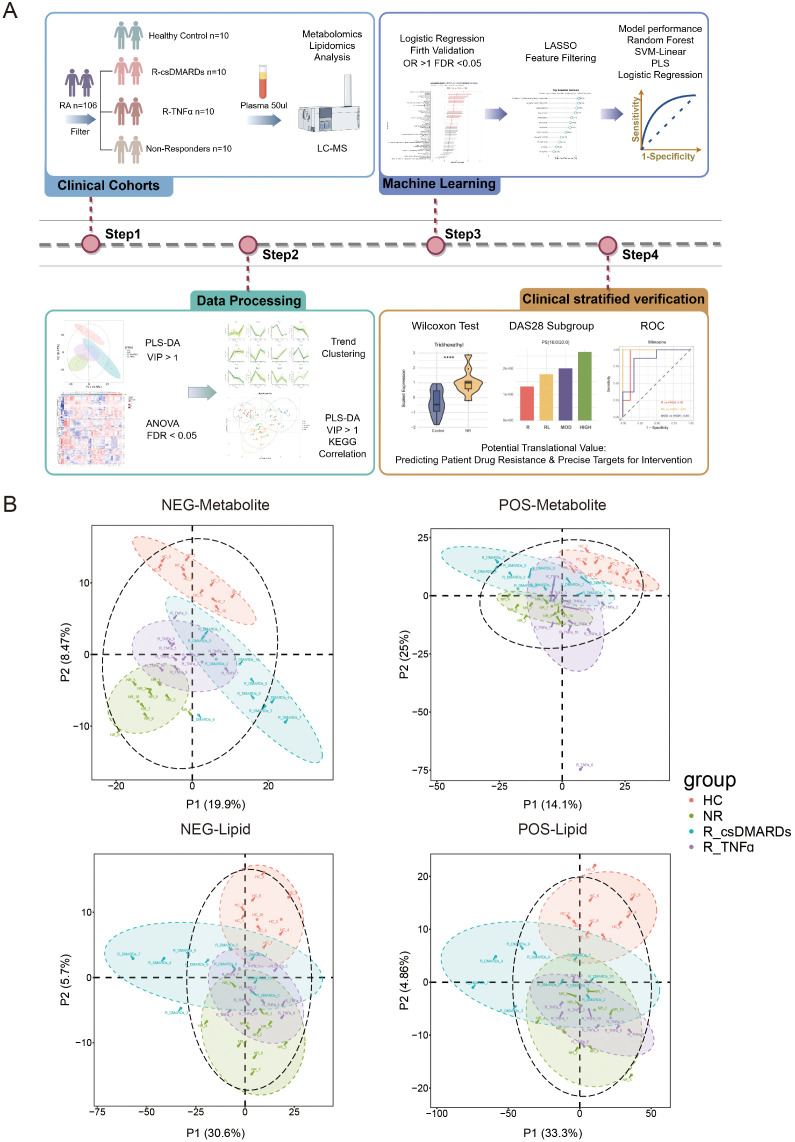
Study workflow and PLS-DA score plots of metabolite/lipid groups. **(A)** Illustrates the workflow of a study integrating clinical cohorts and machine learning to distinguish between patients’ treatment responses. Step 1 involves clinical cohort preparation, followed by Step 2 for data processing which includes PLS-DA analysis and trend clustering. Step 3 applies machine learning techniques such as logistic regression and feature filtering to develop models. Step 4 involves clinical stratified verification using tests like the Wilcoxon test and ROC analysis to validate model performance. **(B)** presents PLS-DA score plots for different metabolite groups, distinguishing between NEG-Metabolite, POS-Metabolite, NEG-Lipid, and POS-Lipid. The percentage values indicating the contribution of each principal component to the data variance. HC, healthy controls; R_csDMARDs, conventional disease-modifying antirheumatic drug therapy response group; R_TNFα, tumor necrosis factor α inhibitor therapy response group; NR, non-response group.

The overall workflow of this study is outlined in **Figure 1A**. Blood samples were obtained from 106 patients diagnosed with RA, excluding individuals with osteoarthritis, infectious diseases, malignant tumors, or other autoimmune disorders. Ultimately, 30 plasma samples from RA patients were selected for analysis based on clinical manifestations and treatment regimens. The clinical characteristics of these participants are summarized in **Table 1**. Based on their treatment modalities and response to therapy, participants were classified into three groups (1): patients receiving conventional disease-modifying anti-rheumatic drugs (csDMARDs) who achieved clinical remission or exhibited low disease activity were categorized as R-csDMARDs (2); patients treated with TNF-α inhibitors who achieved clinical remission or exhibited low disease activity were designated as R-TNFα; and (3) patients receiving at least one DMARD yet experiencing moderate to severe disease activity were categorized as NR (non-response) (27). The grouping of these 30 RA patients based on Disease Activity Score-28 (DAS28) is presented in **Supplementary Table 1**.

**Table 1:** Clinical data of participants.

Group	R-csDMARDs (n=10)	R-TNFα (n=10)	NR(n=10)	HC (n=10)
Demographics				
Female/Male	8/2	3/7	7/3	5/5
Age, Median [Q1, Q3]	54.50 [46.25, 61.75]	51.00 [37.50, 57.00]	60.00 [57.00, 67.75]	55[47,61]
Laboratory parameters, Median [Q1, Q3]				
C-reactive protein (CRP, mg/L)	0.40 [0.40, 0.50]	1.55 [1.07, 2.93]	22.35 [9.50, 84.95]	
Erythrocyte sedimentation rate (ESR, mm/h)	10.00 [5.00, 12.75]	11.00 [5.00, 14.00], n=9	52.50 [40.25, 60.50]	
Rheumatoid factor (RF, IU/mL)	33.05 [15.55, 61.20], n=8	10.10 [6.15, 67.75], n=6	147.25 [83.05, 296.12], n=6	
DAS28-CRP	1.61 [1.32, 2.58]	3.075 [2.54, 4.19]	5.84 [5.28, 6.39]	
Remission (< 2.6), n (%)	8(80.0%)	4(40.0%)	/	
Low disease activity (2.6 ≤ , < 3.2), n (%)	2(20.0%)	2(20.0%)	/	
Moderate disease activity (3.2 < , ≤ 5.1), n (%)	/	4(40.0%)	2(20.0%)	
High disease activity (> 5.1 ), n (%)	/	/	8(80.0%)	
Disease duration (Years)	2.00 [0.62, 5.50]	4.00 [1.50, 9.00]	5.00 [2.25, 9.25]	
Therapeutic agent user, n (%)				
Methotrexate	4(40.0%)	/	3(30.0%)	
Leflunomide	3(30.0%)	/	2(20.0%)	
Hydroxychloroquine sulfate	3(30.0%)	/	1(10.0%)	
Etanercept	/	7(70.0%)	1(10.0%)	
Adalimumab	/	3(30.0%)	3(30.0%)	

Q1, 1st quartile; Q3, 3rd quartile; DAS28, Disease Activity Score in 28 joints; HC, healthy controls (1). patients receiving conventional disease-modifying anti-rheumatic drugs (csDMARDs) who achieved clinical remission or exhibited low disease activity were categorized as R-csDMARDs (2); patients treated with TNF-α inhibitors who achieved clinical remission or exhibited low disease activity were designated as R-TNFα; and (3) patients receiving at least one DMARD yet experiencing moderate to severe disease activity were categorized as NR (non-response.)

Using LC-MS-based untargeted metabolomics and lipidomics analysis, a total of 1,663 metabolites were tentatively identified in positive ion mode (POS) and 616 metabolites in negative ion mode (NEG) after data processing. These compounds included alkaloids, lipids, organic acids, and organic nitrogen compounds. In the lipidomics analysis, 1,977 lipid species were tentatively identified in POS and 1,010 lipid species in NEG, encompassing fatty acids, glycerolipids, and glycerophospholipids. Quantitative analysis of each plasma sample and quality control sample indicated no outliers, confirming that all samples were appropriate for subsequent analysis. The quantitative results of plasma and quality control samples are presented in **Supplementary Figure 2A**.

The partial least squares discriminant analysis (PLS-DA) score plot for plasma metabolomics demonstrated clear separation between RA patients and HC (**Figure 1B**). In the metabolomics analysis, the predictive ability (Q2) was found to be 0.657 for NEG and 0.535 for POS. Although the lipidomics results were less pronounced than those from the metabolomics analysis, the PLS-DA score plot still effectively distinguished between RA patients and healthy controls (**Figure 1B**), with Q2 values of 0.341 for NEG and 0.316 for POS. **Supplementary Figure 2B** provides the evaluation parameters of the PLS-DA models.

To conduct a differential analysis among the four groups, analysis of variance (ANOVA) was employed to assess significant differences, followed by false discovery rate (FDR) correction. A comprehensive analysis revealed that for metabolites with a variable importance in projection (VIP) score greater than or equal to 1 in the PLS-DA and an FDR of less than 0.05, there were 172 metabolites identified in NEG mode and 312 metabolites in POS mode. In the case of lipidomics, 51 lipid species in NEG mode and 138 lipid species in POS mode were identified. The results of the differential analysis, including FDR corrections and VIP scores, are presented in **Supplementary Tables S2A-S2D**. These findings highlight the distinct metabolic and lipidomic profiles in RA patients compared to HC.

### Analysis of differential substances and relationship with disease progression

**Figure 2:**
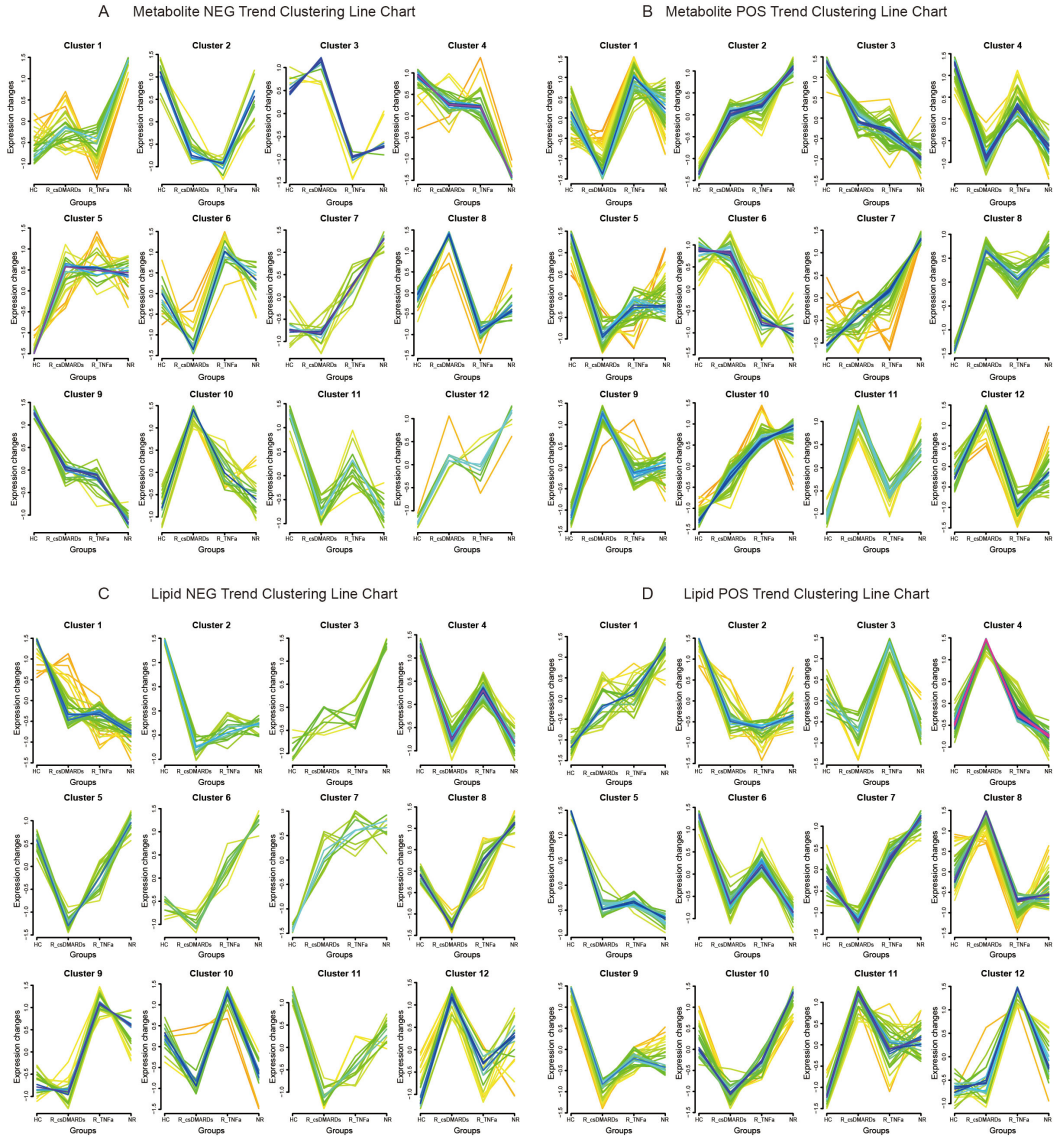
The trend clustering line charts for both metabolite and lipid profiles across different clusters. **(A)** shows the NEG trend clustering line chart for metabolites. **(B)** presents the POS trend clustering line chart for metabolites. **(C, D)** depict the NEG and POS trend clustering line charts for lipids, respectively. Each chart is divided into 12 clusters. The x-axis represents different groups, while the y-axis indicates the expression change.

Further combined analysis of the substances exhibiting statistical significance (VIP score > 1 and FDR < 0.05) revealed that these differential metabolites displayed varying trends with disease progression. Notably, several metabolite clusters showed an increasing trend as the disease advanced: NEG metabolites in Clusters 1, 7, and 12; POS metabolites in Clusters 2, 7, and 10; NEG lipid species in Clusters 3, 6, and 7; and POS lipid species in Clusters 1 and 7 (**Figure 2**). The identified metabolites predominantly included categories such as benzenoids and organoheterocyclic compounds, while the lipid species were primarily classified as glycerophospholipids (GP) and sphingolipids (SP). **Supplementary Figure 3** provides a detailed overview of the metabolite and lipid categories as well as their group-specific expression.

**Figure 3:**
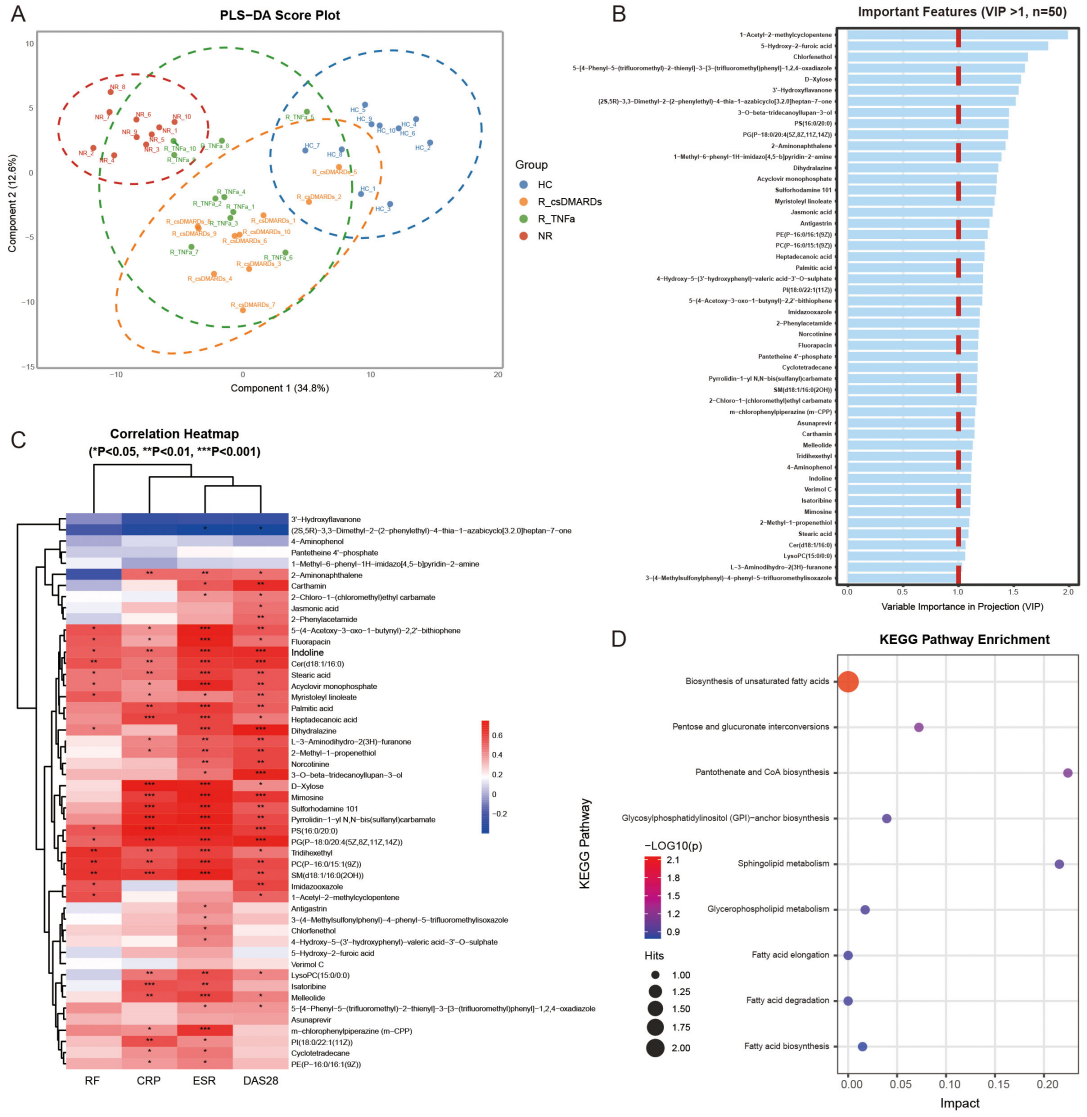
PLS-DA clustering, key features, correlations, and KEGG pathway analysis. **(A)** PLS-DA score plot showing distinct clustering among groups. Component 1 explains 34.8% of the variance. **(B)** Bar chart of important features with VIP scores. **(C)** Correlation heatmap displaying significant correlations (*p<0.05, **p<0.01, ***p<0.001) among various metabolites. **(D)** KEGG pathway analysis showing the impact on different pathways, with bubble size representing the number of hits and color indicating the -LOG(10p) value.

The combined analysis of these clusters indicated that the PLS-DA score plot effectively distinguished among the R group (comprising R-csDMARDs and R-TNFα), HC, and NR (**Figure 3A**), with a Q2 predictive ability of 0.594. **Supplementary Figure 4** provides the evaluation parameters of the PLS-DA models. Additionally, a total of 50 substances with VIP scores greater than 1 were identified within the PLS-DA framework (**Figure 3B**), most of which exhibited significant positive correlations with clinical inflammatory indicators, such as C-reactive protein (CRP) and erythrocyte sedimentation rate (ESR) (**Figure 3C**). Additionally, KEGG pathway enrichment analysis of these substances revealed disrupted metabolic pathways, including the biosynthesis of unsaturated fatty acids, the pentose and glucuronate interconversions, and the pantothenate and coenzyme A (CoA) biosynthesis (**Figure 3D**).

### Diagnostic potential of biomarkers to identify RA treatment non-response

**Figure 4:**
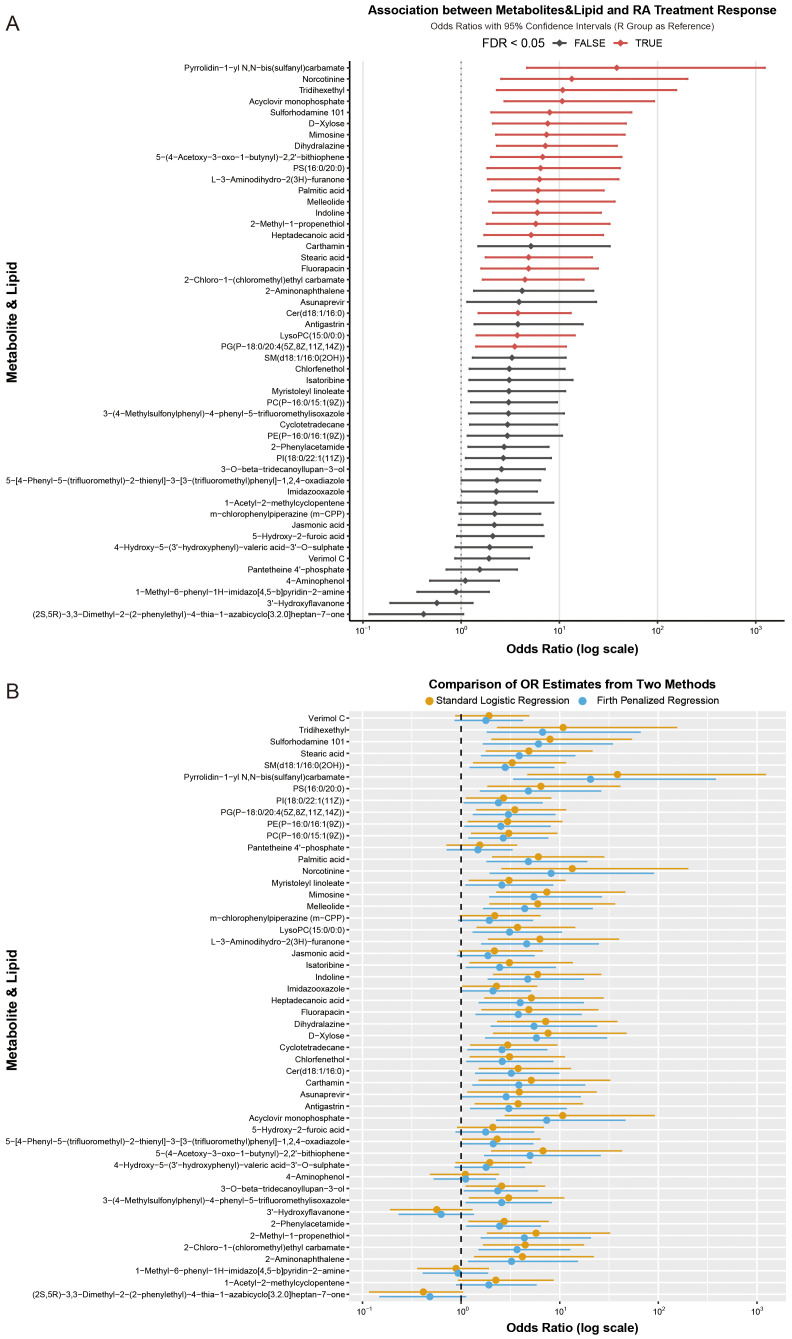
Association between metabolites/lipids and RA treatment response, and OR estimate comparison. **(A)** Forest plot showing the association between metabolites and lipids and RA treatment response, with odds ratios and 95% confidence intervals. A FDR < 0.05 indicates significance. **(B)** Comparison of OR estimates from two methods: standard logistic regression and Firth penalized regression, depicted on a log scale.

Further analysis of the substances exhibiting a VIP score greater than 1 (totaling 50) was conducted to assess their potential in diagnosing treatment non-response RA. Logistic regression applied to the three groups of RA data identified 22 metabolites and lipids strongly associated with RA risk, all of which exhibited OR greater than 1. Following FDR correction, the p-values for all substances remained below 0.05 (**Figure 4A**). OR values derived from Firth penalized regression showed no significant bias (**Figure 4B**), confirming the stability and reliability of these findings.

**Figure 5:**
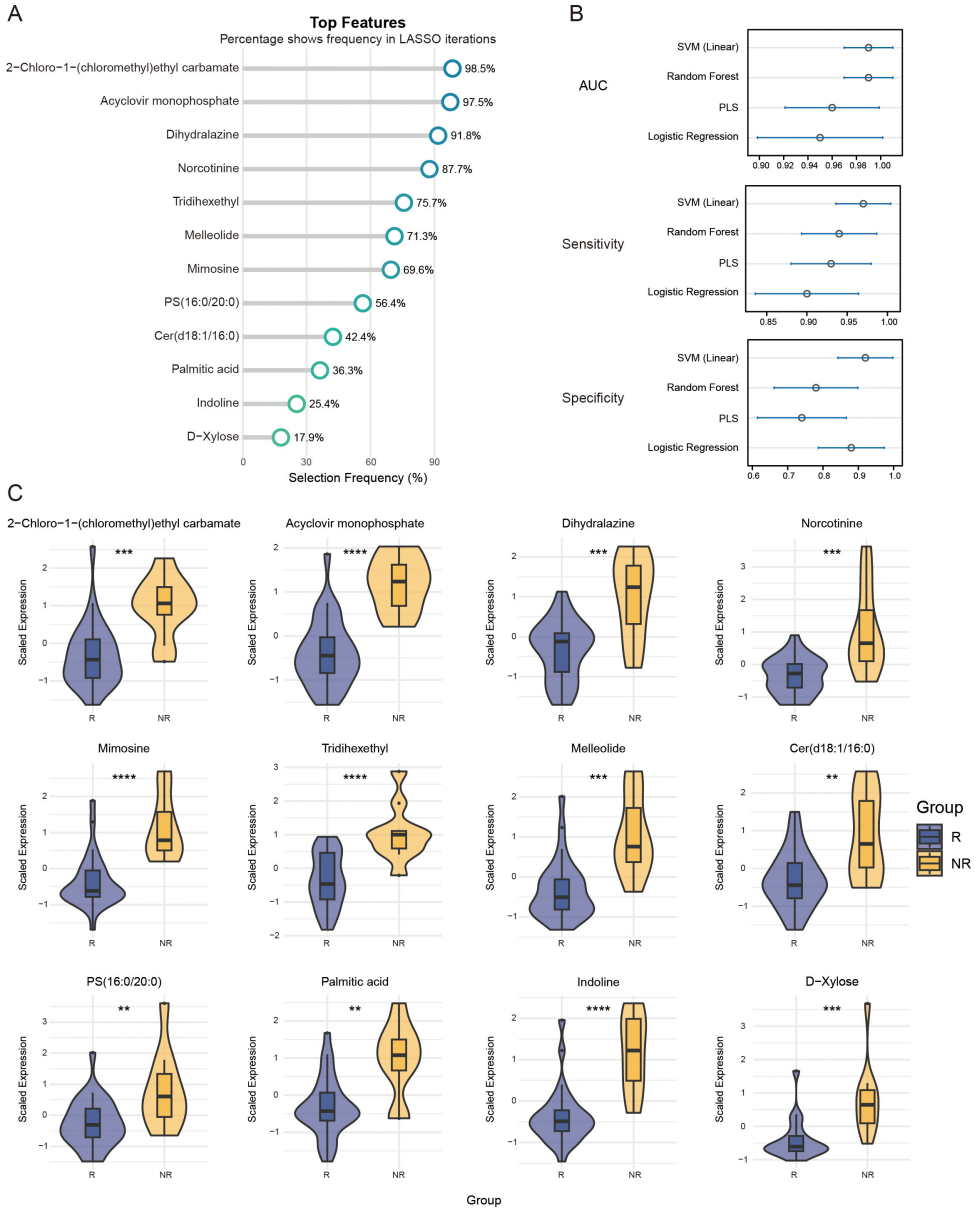
LASSO feature importance, machine learning model performance, and key biomarker expression. **(A)** Top feature importance based on frequency in LASSO iterations. **(B)** Model performance metrics including AUC, sensitivity, and specificity for different machine learning methods. **(C)** Violin plots showing scaled expression levels of key metabolites and lipids, comparing R and NR, with significance levels indicated by asterisks(*p<0.05, **p<0.01, ***p<0.001, ****p<0.0001).

Subsequently, we employed LASSO regularization to further refine this selection of 22 substances, ultimately identifying 12 core features: 2-Chloro-1-(chloromethyl)ethyl carbamate, acyclovir monophosphate, dihydralazine, norcotinine, mimosine, tridihexethyl, melleolide, palmitic acid, indoline, D-xylose, along with the lipids Cer(d18:1/16:0) and PS(16:0/20:0) (**Figure 5A**). To preliminarily explore the predictive capability of these substances for treatment outcomes, we internally constructed and cross-validated a machine learning model based on these 12 core features. The AUC achieved using SVM (Linear), Random Forest, PLS, and Logistic Regression (with 10×5-fold cross-validation) consistently exceeded 0.90 in internal cross-validation, suggesting promising preliminary discriminatory capability within this limited study cohort. Both specificity and sensitivity measures were greater than 0.70 and 0.90, respectively (**Figure 5B**). Additionally, Wilcoxon rank-sum tests revealed a significant difference in the expression of these twelve substances between the R group (including R-csDMARDs and R-TNFα) and the NR group, with p-values all less than 0.01, and all substances being significantly more highly expressed in the NR group (**Figure 5C**). These preliminary results highlight the tentative potential of these candidate biomarkers in distinguishing RA treatment responders from NR, and relevant findings should be interpreted cautiously given the relatively small sample size.

**Figure 6:**
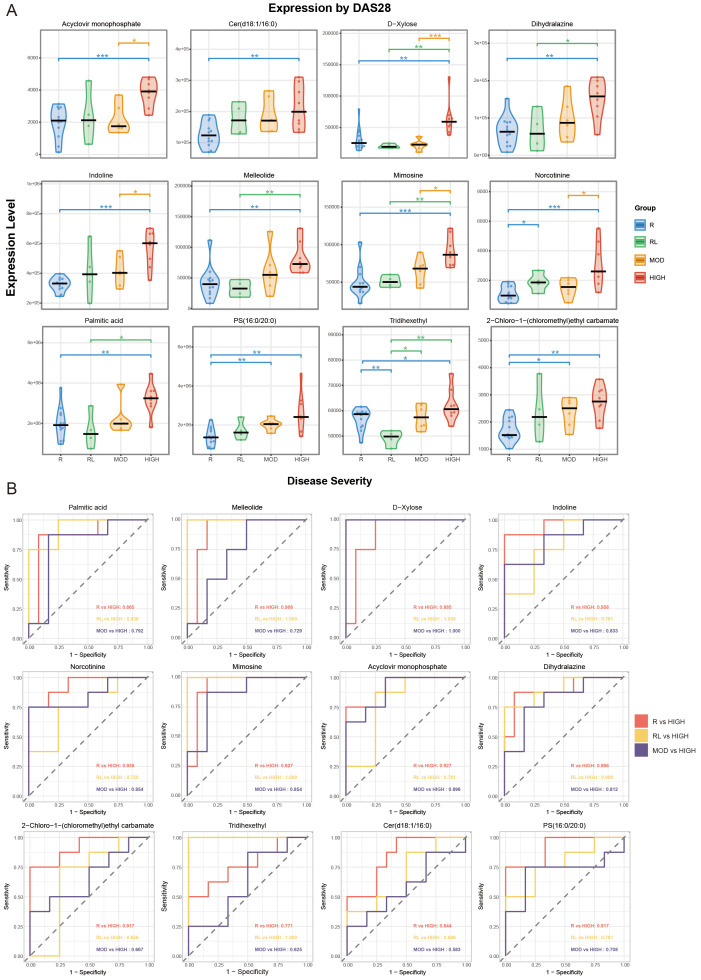
Metabolite and lipid expression levels across disease activity (DAS28) groups and ROC curves for subjects across groups. **(A)** The expression levels of key metabolites and lipids across different group: R (Remission), RL (Low disease activity), MOD (Moderate disease activity), and HIGH (High disease activity). **(B)** ROC curve analysis comparing the performance of various metabolites and lipids in distinguishing between different response groups, with the AUC indicated for each. Intergroup differences were assessed using the Mann-Whitney U test, with P values adjusted for FDR. *p<0.05, **p<0.01, ***p<0.001, ****p<0.0001. Remission: DAS28 Score < 2.6, Low disease activity: 2.6 ≤ DAS28 Score < 3.2, Moderate disease activity: 3.2 < DAS28 Score ≤ 5.1, High disease activity: DAS28 Score > 5.1.

By stratifying the RA patients based on their DAS28 scores, participants were classified into remission (R), low disease activity (RL), moderate disease activity (MOD), and high disease activity (HIGH) groups. The expression levels of the tentatively annotated metabolites and lipids were analyzed across these groups (**Figure 6**). Notably, all 12 core features showed significantly higher expression in the HIGH group compared to the R group (p < 0.01). Furthermore, metabolites and lipids such as dihydralazine, indoline, Cer(d18:1/16:0), 2-Chloro-1-(chloromethyl)ethyl carbamate, and PS(16:0/20:0) exhibited progressively increasing levels with higher disease activity (**Figure 6A**), showing a potential trend for discriminating different disease activity stages within this cohort. Specifically, the area under the curve (AUC) for dihydralazine in distinguishing the HIGH group from RL was 0.906, while its AUC against the MOD group was 0.812 (**Figure 6B**). Similarly, indoline showed promising discriminatory performance, with an AUC of 0.958 in distinguishing HIGH from R, and 0.833 against the MOD group (**Figure 6B**). Additionally, metabolites such as palmitic acid, acyclovir monophosphate, D-xylose, norcotinine, mimosine, Tridihexethyl, and melleolide were also highly expressed in the HIGH group (**Figure 6A**). Of particular importance, D-xylose and melleolide presented extremely high AUC values in this small sample cohort, with AUC values of 1 for comparisons between the HIGH and RL groups, as well as between the HIGH and MOD groups for D-xylose, and the HIGH and RL groups for melleolide (**Figure 6B**). These preliminary findings suggest the potential utility of these candidate biomarkers in distinguishing disease severity and assessing treatment efficacy in RA. However, it should be noted that the observed phenomenon of AUC = 1 for D-xylose and melleolide may be influenced by the relatively small sample size, which warrants cautious interpretation and requires further validation in larger independent cohorts.

## Discussion

This study stratified RA patients based on their treatment responses, with a particular focus on those exhibiting inadequate therapeutic outcomes. Through a comprehensive analysis of plasma metabolites and lipids, tentatively annotated biochemical alterations associated with treatment non-response in RA were identified. Multiple statistical methods, including differential analysis, PLS-DA, LASSO, and logistic regression, were applied to pinpoint 12 metabolites and lipids potentially correlated with disease progression and therapeutic outcomes. Using these candidate molecular features, a predictive model was constructed and internally cross-validated, showing promising preliminary performance with an area under the receiver operating characteristic curve (AUC-ROC) exceeding 0.90 in the present small sample cohort. Notably, these features demonstrated significant correlations with disease activity, suggesting tentative potential as candidate biomarkers for evaluating therapeutic response in RA patients.

Among the identified biomarkers, two lipids—Cer(d18:1/16:0) and PS(16:0/20:0)—were significantly correlated with RA disease activity. Their expression levels increased proportionally with disease severity, consistent with previous findings that linked elevated Cer levels to RA disease severity (28). Cer(d18:1/16:0) has been associated with atherosclerosis (29), whereas PS(16:0/20:0) has been implicated in coagulation processes (30, 31). Both lipids share a structural feature—16:0 (palmitic acid)—which was also identified in this study as being associated with disease severity. Other studies have reported that palmitic acid exacerbates RA progression by promoting the secretion of inflammatory mediators via pyroptosis activation through the NLRP3/Caspase1/GSDMD-N/IL-1β pathway (32).

Two metabolites, mimosine and D-xylose, were found to be associated with treatment non-response in RA. Mimosine, a plant-derived amino acid, functions as a normoxic inducer of hypoxia-inducible factor (HIF) (33). Previous research has demonstrated its dual role in disrupting bone metabolism homeostasis, through both inhibition of osteoblast synthesis of osteoprotegerin (a key protective factor for bone integrity) and promotion of osteoclast formation via the activation of the HIF1α-MAPK pathway (33, 34). These mechanisms collectively contribute to enhanced bone resorption and disease progression. In addition, elevated levels of D-xylose were observed in patients with treatment non-response. Supplementation of D-xylose *in vivo* has been reported to increase CD8+ T cell infiltration and upregulate cytotoxic marker expression (35), potentially contributing to the observed resistance to therapeutic interventions.

In this study, the pathways Biosynthesis of Unsaturated Fatty Acids, Pentose and Glucuronate Interconversion, and Pantothenate and CoA Biosynthesis were enriched in the treatment non-response patient group. These pathways have also been previously reported as key contributors to the pathophysiology of RA (32). Among the metabolites identified in our study, D-xylose is likely a product of the glucuronate pathway within the pentose and glucuronate interconversion pathway. In RA, impaired intestinal barrier function and dysbiosis, coupled with increased intestinal permeability (36), may facilitate the absorption of bacterial metabolites, including D-xylose, into the bloodstream, thereby activating the pentose and glucuronate interconversion pathway. Furthermore, under inflammatory conditions, enhancement of the pantothenate and CoA biosynthesis pathway likely sustains CoA production via pantothenate metabolism. Adequate CoA availability subsequently enables the activation of saturated fatty acids, such as palmitic acid, into palmitoyl-CoA, which serves as the critical upstream substrate for the Biosynthesis of Unsaturated Fatty Acids pathway. These findings align with growing evidence of interconnectivity among metabolic dysfunction, inflammation, and disease progression in RA.

Several metabolites associated with drug metabolism were identified, including indoline, dihydralazine, tridihexethyl, melleolide and acyclovir monophosphate. These substances were found to be highly expressed in the treatment non-response group and were predominantly associated with higher disease activity. Indoline is believed to be derived from metabolism of indole-containing drugs, such as indomethacin, a common nonsteroidal anti-inflammatory drug. Armillaria mellea, an herb widely used in traditional Chinese medicine, is believed to help “dispel wind, activate meridians, and strengthen bones and muscles.” Melleolide, a unique active compound found in the mycelium of Armillaria mellea (37). Dihydralazine, tridihexethyl, and acyclovir monophosphate were further associated with extra-articular manifestations, such as cardiovascular (38, 39), gastrointestinal (40), and herpesvirus-related conditions (41), respectively. Extra-articular manifestations are known to correlate with poor prognosis and increased mortality in RA (42, 43). Therefore, it is hypothesized that these metabolic features may serve as critical metabolic bridges, connecting treatment non-response, extra-articular manifestations, and adverse outcomes in RA.

Among the metabolites identified to be associated with treatment non-response, norcotinine and 2-chloro-1-(chloromethyl)ethyl carbamate were found to be linked to exposure to air pollution. Norcotinine, a metabolite of nicotine, has been associated with both active and passive smoking—recognized risk factors for RA with established associations to increased disease activity (44, 45). The metabolite 2-chloro-1-(chloromethyl)ethyl carbamate is hypothesized to be related to exposure to chlorinated air pollutants. Extensive studies have demonstrated a strong association between long-term cumulative exposure to environmental air pollutants and the risk of RA (46, 47).

In addition to the findings discussed above, a total of 2,279 metabolites and 2,987 lipids were tentatively annotated in this study, far exceeding the number reported in most similar studies to date. This provides a more comprehensive dataset for advancing research in RA. Despite the encouraging results, certain limitations of this study must be acknowledged. Notably, the small sample size of healthy controls for baseline comparison may introduce potential analytical bias and reduce the representativeness of normal molecular reference profiles. The relatively small sample size may have led to overestimation or underestimation of effect sizes for some potential biomarkers, particularly for metabolites with low abundance but significant pathological relevance, whose true associations may not have been accurately captured due to the limited number of samples. Moreover, the sample size was insufficient to fully account for the heterogeneity among RA patients, such as differences in age stratifications, disease stages, treatment histories, and coexisting conditions. This limitation may restrict the generalizability of the identified biomarkers, making them primarily applicable to the specific patient subgroup included in this study while limiting their utility for broader RA populations. Furthermore, the machine learning-based prognostic diagnostic model established in this study only underwent internal validation within the 30 RA patient samples, without the support of an independent external validation cohort. Compared with external validation, internal validation is more susceptible to overfitting risks. In addition, with the continuous advancement and evolution of therapeutic strategies for RA, the metabolomic signatures of treatment non-responders are likely to change dynamically over time. Accordingly, the molecular profiles identified in the present study should not be regarded as a definitive and unaltered conclusion.

Despite the above inherent limitations, we openly report and release the comprehensive metabolomic and lipidomic data generated in this work. These raw and annotated omics resources can serve as a valuable reference for subsequent related investigations, providing accessible data support for further exploring molecular mechanisms and screening predictive biomarkers in RA treatment non-response. Nevertheless, our findings still provide valuable clues for the early recognition of RA patients at high risk of poor treatment response. To overcome these challenges, future research with larger sample sizes and multi-cohort investigations involving diverse RA populations is essential. Moreover, well−designed prospective clinical studies are warranted to further validate the predictive value of the identified differential metabolites for treatment response. This would allow the identification of more universally applicable biomarkers related to treatment response in RA, enhancing the generalizability of the findings and providing more robust evidence to support clinical diagnosis, treatment, and prevention strategies for RA.

## Conclusions

In summary, this study provides comprehensive insights into the plasma metabolomics and lipidomics profiles associated with RA, underscoring the potential of specific metabolites and lipids to serve as biomarkers for diagnosing treatment non-response. A total of 2,279 metabolites and 2,987 lipids were tentatively annotated. Through rigorous statistical evaluation (VIP > 1 and FDR < 0.05), 22 metabolites and lipids were found to be positively associated with RA risk based on logistic regression analysis. These were subsequently refined into 12 core features using LASSO regression. The 12 core features identified in the treatment non-response group include lipids Cer(d18:1/16:0), PS(16:0/20:0), and Palmitic acid, metabolites Mimosine and D-Xylose, drug-related metabolites Dihydralazine, Tridihexethyl, Acyclovir monophosphate, Indoline, and Melleolide, as well as pollution-related metabolites Norcotinine and 2-Chloro-1-(chloromethyl)ethyl carbamate. Machine learning models employing these features showed promising preliminary discriminatory performance, achieving AUC values exceeding 0.90, in internal cross-validation within the limited sample cohort, suggesting tentative potential for distinguishing treatment-responsive and non-responsive RA patients. Additionally, analysis of biomarker expression across disease activity levels (remission, low, moderate, and high disease activity) revealed that the majority of these biomarkers were significantly associated with high disease activity. Overall, the findings provide preliminary insights into metabolic and lipid alterations underlying RA pathophysiology and imply tentative application potential of these tentatively annotated candidate biomarkers in predicting treatment responses and optimizing personalized therapeutic strategies. Future studies with larger cohorts are warranted to validate these findings and further investigate the mechanisms underlying the involvement of these metabolites in RA progression and treatment response.

## Data Availability

The metabolomics and lipidomics data generated in this study have been submitted to the MetaboLights repository under the accession numbers MTBLS13243, MTBLS13238. All other supporting data that underpin the findings of this study are included in the **Supplementary Material** of this manuscript.

## Glossary

ACR: American College of Rheumatology
AUC: Area Under the Curve
Caspase1: Cysteine-Aspartic Acid Protease 1
CoA: Coenzyme A
CRP: C-reactive Protein
csDMARDs: Conventional Disease-Modifying Anti-Rheumatic Drugs
DAS28: Disease Activity Score-28
DMARDs: Disease-Modifying Antirheumatic Drugs
EULAR: European League Against Rheumatism
ESR: Erythrocyte Sedimentation Rate
FDR: False Discovery Rate
GSDMD-N: Gasdermin D-N Terminal
GP: Glycerophospholipids
HC: Healthy Controls
HIF: Hypoxia-Inducible Factor
HIGH: High Disease Activity
IL-1β: Interleukin-1β
KEGG: Kyoto Encyclopedia of Genes and Genomes
LC-MS: Liquid Chromatography–Mass Spectrometry
LASSO: Least Absolute Shrinkage and Selection Operator
MAPK: Mitogen-Activated Protein Kinase
MOD: Moderate Disease Activity
NEG: Negative Ion Mode
NLRP3: NOD-like Receptor Family Pyrin Domain Containing 3
NSAIDs: Nonsteroidal Anti-Inflammatory Drugs
NR: Non-Response
OR: Odds Ratio
POS: Positive Ion Mode
PLS: Partial Least Squares
PLS-DA: Partial Least Squares Discriminant Analysis
RA: Rheumatoid Arthritis
R-csDMARDs: Responders to Conventional Disease-Modifying Anti-Rheumatic Drugs
R-TNFα: Responders to Tumor Necrosis Factor-Alpha Inhibitors
RL: Low Disease Activity
ROC: Receiver Operating Characteristic
SP: Sphingolipids
SVM: Support Vector Machine
TNF-α: Tumor Necrosis Factor-Alpha
VIP: Variable Importance in Projection.
