# Supplementary material for: Plasma metabolomic and lipidomic signatures characteristic of treatment non-response in rheumatoid arthritis
Source: Front Immunol. 2026 Jun 4;17:1787287. doi: 10.3389/fimmu.2026.1787287 (PMC13275419; doi:10.3389/fimmu.2026.1787287)
Supplement: Supplementary file 2 [file DataSheet2.pdf]

Supplementary Material

Liquid chromatography – tandem mass spectrometry analysis

Metabolomics Analysis:

Sample preparation involved thawing samples at 4 °C, to which four volumes of pre-chilled methanol/acetonitrile (1:1) were added and vortexed for 30 seconds before being incubated at −20 °C for 1 hour. Samples were then centrifuged at 14,000 g for 20 minutes at 4 °C, and the supernatant was vacuum dried at room temperature. Dried samples were stored at −80 °C. QC samples were prepared by pooling equal volumes and processed similarly.

Chromatographic conditions included mobile phase A (10 mM ammonium acetate in a 95:5 acetonitrile/water solution with 0.1% formic acid) and mobile phase B (10 mM ammonium acetate in a 50:50 acetonitrile/water solution with 0.1% formic acid). The elution gradient started at 2% mobile phase B for 0.5 minutes, increasing to 98% over 11.5 minutes, followed by a 4-minute hold at 98% B.

Mass spectrometry was conducted in both positive and negative modes within a mass scan range of 70–1050 m/z. The first-level mass resolution was set at 120,000 (AGC target: 3e6, max IT: 100 ms), while the second-level resolution was 7,500 (AGC target: 2e5, max IT: 50 ms), utilizing HCD fragmentation.

Lipidomics Analysis:

Sample preparation for lipidomics involved mixing each sample with four volumes of pre-chilled methanol/water (2:1), vortexing, followed by the addition of eight volumes of MTBE, and subjecting the mixture to low-temperature ultrasonication for 30 minutes. The samples were centrifuged at 14,000 g for 10 minutes at 4 °C, separating the upper layer for vacuum drying. Dried samples were stored at −80 °C, and QC samples were prepared in parallel.

For lipidomics chromatography, mobile phase A comprised 10 mM ammonium formate in a 60:40 acetonitrile/water solution with 0.1% formic acid, and mobile phase B consisted of 10 mM ammonium formate in a 90:10 isopropanol/acetonitrile solution with 0.1% formic acid. The elution gradient started at 30% mobile phase B for 20 minutes, gradually increased to 100%, and then returned to 30% B.

Mass spectrometry parameters included a parent ion scanning range of 200 to 2000 m/z. First-stage resolution was set at 120,000, and second-stage resolution at 15,000, utilizing HCD fragmentation at normalized collision energies of 20, 40, and 60.

Supplementary Figures

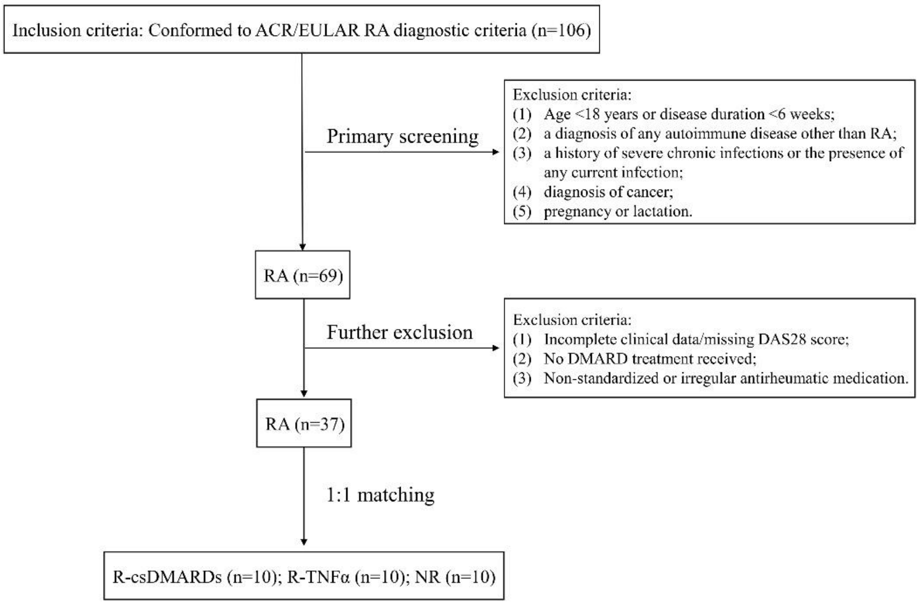

Supplement Figure 1: Flowchart of patient enrollment and screening process. A total of 106 patients with rheumatoid arthritis (RA) who met the American College of Rheumatology (ACR)/European League Against Rheumatism (EULAR) classification criteria were initially included in the study.After primary screening (exclusion of patients aged <18 years, with disease duration <6 weeks, other autoimmune diseases, severe chronic or current infections, malignancy, or pregnancy/lactation), 69 eligible RA patients remained.Further exclusion was conducted to remove patients with incomplete clinical data (including missing Disease Activity Score in 28 joints [DAS28] scores), no history of disease-modifying antirheumatic drug (DMARD) treatment, or non-standardized/irregular antirheumatic medication, resulting in 37 remaining patients.The 37 eligible patients were stratified into three subgroups and underwent 1:1 matching, yielding a final cohort of 30 RA patients (10 patients per group) included in the subsequent omics analyses.

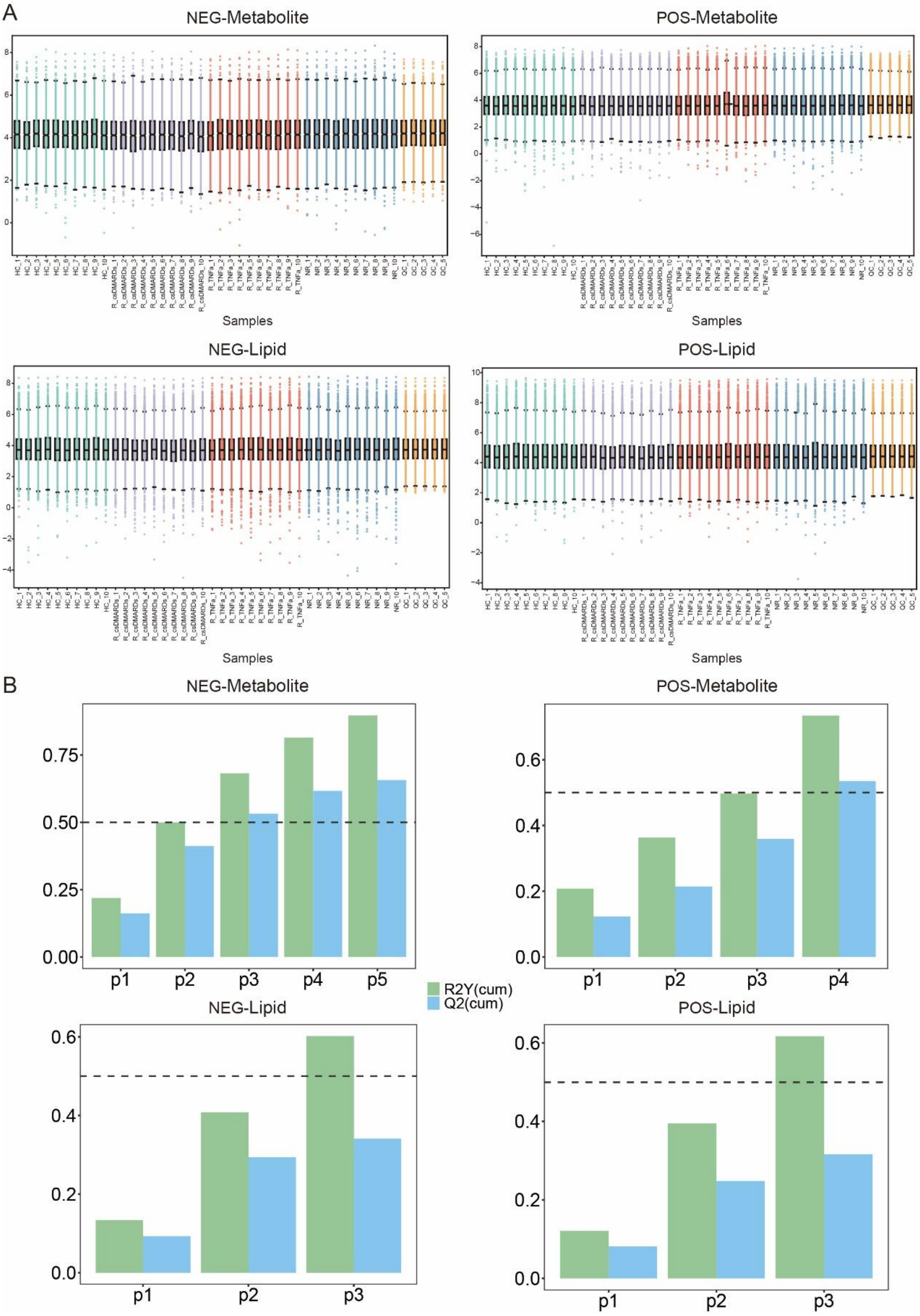

Supplement Figure 2: A: The quantitative results of metabolites identified in experimental samples and QC samples are displayed in a box plot, with the sample name on the horizontal axis and the quantitative results of metabolites (log10) on the vertical axis. The horizontal line in the middle of the box plot represents the median value, which can reflect whether there is a deviation in the overall quantitative values of metabolites between samples. B: Cross-validation is used to obtain model

evaluation parameters (R2Y, Q2), where R2Y is the model's explanatory power for the classification variable Y, and Q2 is the model's predictive power. Generally, when Q2 > 0.5, it indicates that the model is stable and reliable.

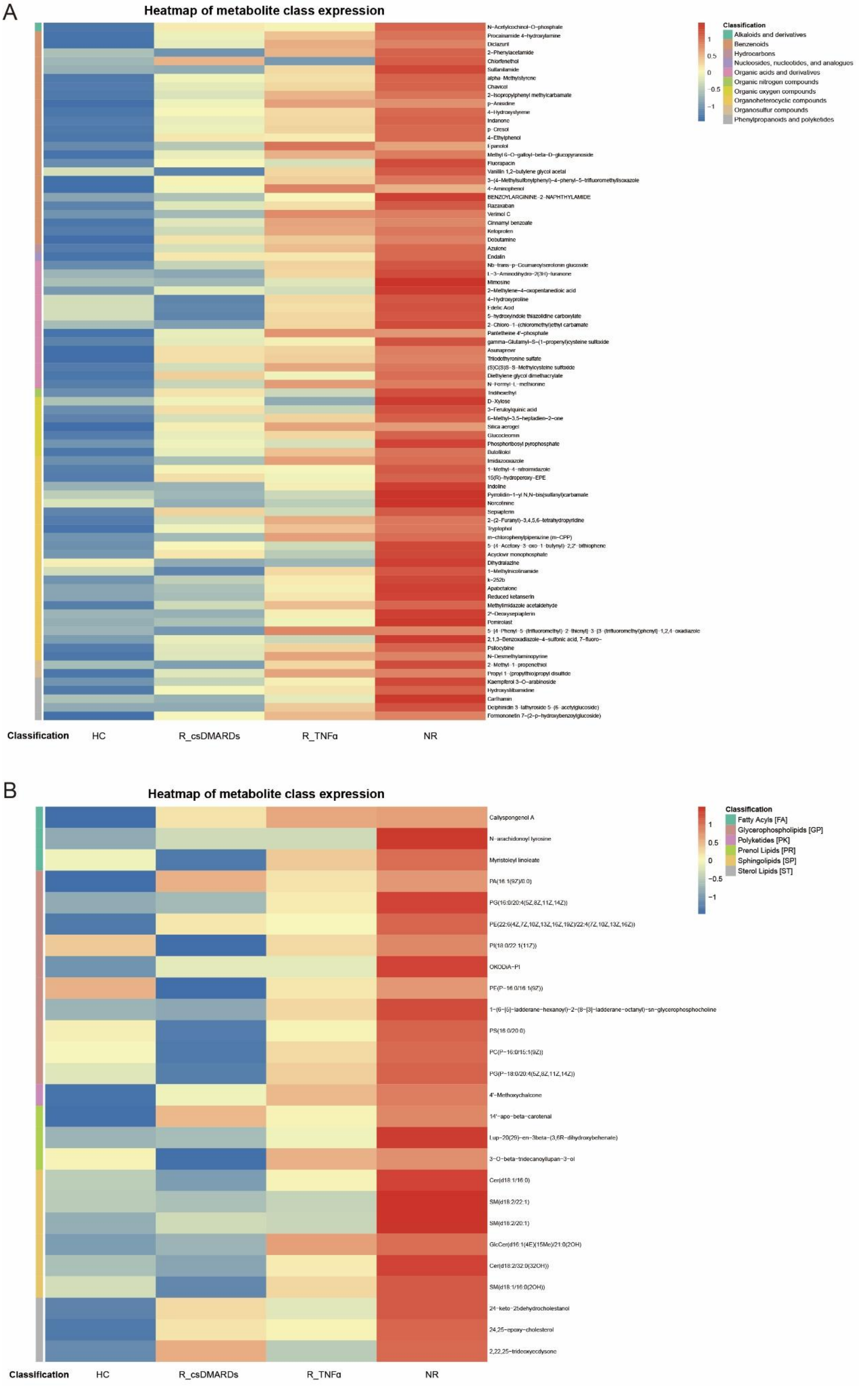
A
Heatmap of metabolite class expression
N−Acetylcochinol−O−phosphate
Procainamide 4−hydroxylamine
Diclazuril
2−Phenylacetamide
Chlorfenethol
Sulfanilamide
alpha−Methylstyrene
Chavicol
2−Isopropylphenyl methylcarbamate
p−Anisidine
4−Hydroxystyrene
Indanone
p−Cresol
4−Ethylphenol
Epanolol
Methyl 6−O−galloyl−beta−D−glucopyranoside
Fluorapacin
Vanillin 1,2−butylene glycol acetal
3−(4−Methylsulfonylphenyl)−4−phenyl−5−trifluoromethylisoxazole
4−Aminophenol
BENZOYLARGININE−2−NAPHTHYLAMIDE
Razaxaban
Verimol C
Cinnamyl benzoate
Ketoprofen
Dobutamine
Azulene
Endalin
Nb−trans−p−Coumaroylserotonin glucoside
L−3−Aminodihydro−2(3H)−furanone
Mimosine
2−Methylene−4−oxopentanedioic acid
4−Hydroxyproline
Edetic Acid
5−hydroxyindole thiazolidine carboxylate
2−Chloro−1−(chloromethyl)ethyl carbamate
Pantetheine 4'−phosphate
gamma−Glutamyl−S−(1−propenyl)cysteine sulfoxide
Asunaprevir
Triiodothyronine sulfate
(S)C(S)S−S−Methylcysteine sulfoxide
Diethylene glycol dimethacrylate
N−Formyl−L−methionine
Indihexethyl
D−Xylose
3−Feruloylquinic acid
6−Methyl−3,5−heptadien−2−one
Silica aerogel
Glucocleomin
Phosphoribosyl pyrophosphate
Butofilolol
Imidazooxazole
1−Methyl−4−nitroimidazole
15(R)−hydroperoxy−EPE
Indoline
Pyrrolidin−1−yl N,N−bis(sulfanyl)carbamate
Norcotinine
Sepiapterin
2−(2−Furanyl)−3,4,5,6−tetrahydropyridine
Tryptophol
m−chlorophenylpiperazine (m−CPP)
5−(4−Acetoxy−3−oxo−1−butynyl)−2,2'−bithiophene
Acyclovir monophosphate
Dihydralazine
1−Methylnicotinamide
k−252b
Apabetalone
Reduced ketanserin
Methylimidazole acetaldehyde
2'−Deoxysepiapterin
Pemirolast
5−[4−Phenyl−5−(trifluoromethyl)−2−thienyl]−3−[3−(trifluoromethyl)phenyl]−1,2,4−oxadiazole
2,1,3−Benzoxadiazole−4−sulfonic acid, 7−fluoro−
Psilocybine
N−Desmethylaminopyrine
2−Methyl−1−propenethiol
Propyl 1−(propylthio)propyl disulfide
Kaempferol 3−O−arabinoside
Hydroxystilbamidine
Carthamin
Delphinidin 3−lathyroside 5−(6−acetylglucoside)
Formononetin 7−(2−p−hydroxybenzoylglucoside)
Classification
Alkaloids and derivatives
Benzenoids
Hydrocarbons
Nucleosides, nucleotides, and analogues
Organic acids and derivatives
Organic nitrogen compounds
Organic oxygen compounds
Organoheterocyclic compounds
Organosulfur compounds
Phenylpropanoids and polyketides
1
0.5
0
−0.5
−1
Classification
HC
R_csDMARDs
R_TNFα
NR
B
Heatmap of metabolite class expression
Callyspongenol A
N−arachidonoyl tyrosine
Myristoleyl linoleate
PA(16:1(9Z)/0:0)
PG(16:0/20:4(5Z,8Z,11Z,14Z))
PE(22:6(4Z,7Z,10Z,13Z,16Z,19Z)/22:4(7Z,10Z,13Z,16Z))
PI(18:0/22:1(11Z))
OKODiA−PI
PE(P−16:0/16:1(9Z))
1−(6−[5]−ladderane−hexanoyl)−2−(8−[3]−ladderane−octanyl)−sn−glycerophosphocholine
PS(16:0/20:0)
PC(P−16:0/15:1(9Z))
PG(P−18:0/20:4(5Z,8Z,11Z,14Z))
4'−Methoxychalcone
14'−apo−beta−carotenal
Lup−20(29)−en−3beta−(3,6R−dihydroxybehenate)
3−O−beta−tridecanoyllupan−3−ol
Cer(d18:1/16:0)
SM(d18:2/22:1)
SM(d18:2/20:1)
GlcCer(d16:1(4E)(15Me)/21:0(2OH)
Cer(d18:2/32:0(32OH))
SM(d18:1/16:0(2OH))
24−keto−25dehydrocholestanol
24,25−epoxy−cholesterol
2,22,25−trideoxyecdysone
Classification
Fatty Acyls [FA]
Glycerophospholipids [GP]
Polyketides [PK]
Prenol Lipids [PR]
Sphingolipids [SP]
Sterol Lipids [ST]
1
0.5
0
−0.5
−1
Classification
HC
R_csDMARDs
R_TNFα
NR

Supplement Figure 3: A: Heatmap of metabolite class expression. B: Heatmap of lipid class expression. The vertical axis shows the names of metabolites and lipids and their categories, the horizontal axis shows the grouping.

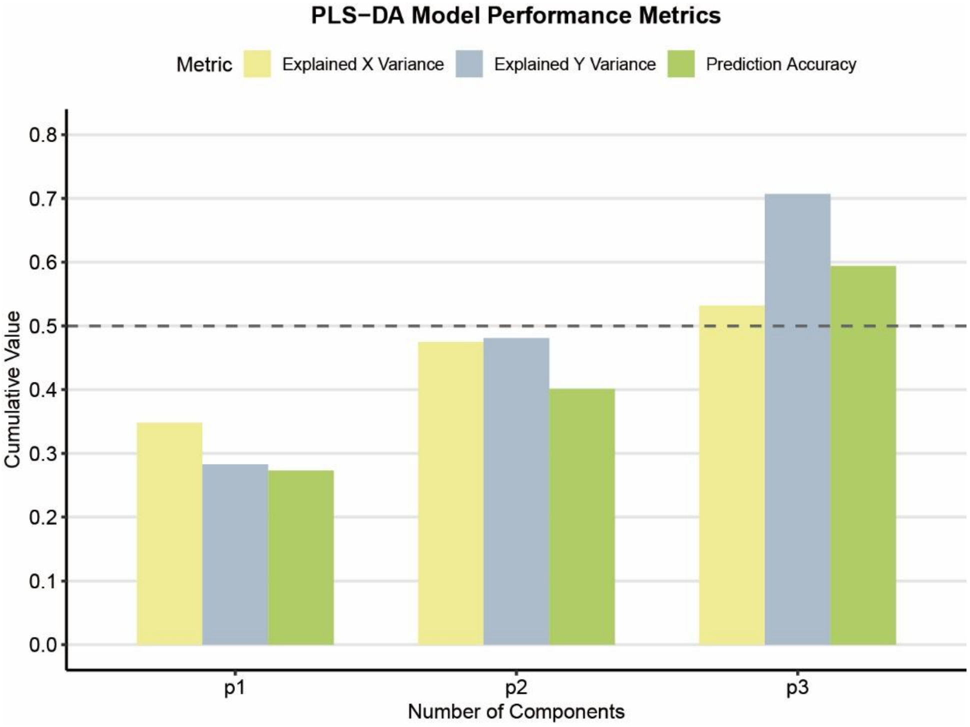

Supplement Figure 4: Performance Metrics of the PLS-DA Model Across Different Component Levels. The metrics evaluated include Explained X Variance (yellow bars), Explained Y Variance (blue bars), and Prediction Accuracy (green bars). The dashed line at 0.5 indicates the threshold value for Prediction Accuracy.

References

[1] Nicholson, Jeremy K et al. "Metabonomics: a platform for studying drug toxicity and gene function." Nature reviews. Drug discovery vol. 1,2 (2002): 153-61. doi:10.1038/nrd728

[2] Rinschen MM. et al. Identification of bioactive metabolites using activity metabolomics: Nat Rev Mol Cell Biol. 353-367(2019).

[3] Jang C. et al. Metabolomics and Isotope Tracing. Cell. 822-837(2018).

[4] Johnson CH. et al. Metabolomics: beyond biomarkers and towards mechanisms. Nat Rev Mol Cell Biol. 449-451(2016).

[5] Leao TF. Et al. Quick-start infrastructure for untargeted metabolomics analysis in GNPS. Nat Metab. 880-882(2021).

[6] Chen L. et al. Metabolite discovery through global annotation of untargeted metabolomics data. Nat Methods. 1377-1385(2021).

[7] Bauermeister A. et al. Mass spectrometry-based metabolomics in microbiome investigations. Nat Rev Microbiol. 143-160(2022).

[8] Liang, Liang et al. "Metabolic Dynamics and Prediction of Gestational Age and Time to Delivery in Pregnant Women." Cell vol. 181,7 (2020): 1680-1692.e15. doi:10.1016/j.cell.2020.05.002

[9] Huang, Yida et al. "Diagnosis and prognosis of breast cancer by high-performance serum metabolic fingerprints." Proceedings of the National Academy of Sciences of the United States of America vol. 119,12 (2022): e2122245119. doi:10.1073/pnas.2122245119.
